# The Impact of *ABCC2 -24C>T* Gene Polymorphism on Graft Survival in Kidney Transplant Recipients

**DOI:** 10.3390/jpm14040440

**Published:** 2024-04-22

**Authors:** Chiau Ling Choong, Farida Islahudin, Hin-Seng Wong, Rosnawati Yahya, Nor Asyikin Mohd Tahir, Mohd Makmor-Bakry

**Affiliations:** 1 Center of Quality Medicine Management, Faculty of Pharmacy, Universiti Kebangsaan Malaysia, Jalan Raja Muda Abdul Aziz, Kuala Lumpur 50300, Malaysia; p112748@siswa.ukm.edu.my (C.L.C.); syikin.tahir@ukm.edu.my (N.A.M.T.); mohdclinpharm@ukm.edu.my (M.M.-B.); 2 Department of Nephrology, Selayang Hospital, Ministry of Health Malaysia, Batu Caves 68100, Malaysia; hinseng@gmail.com; 3 Sunway Medical Centre, Jalan Lagoon Selatan, Bandar Sunway, Subang Jaya 46150, Malaysia; 4 Department of Nephrology, Kuala Lumpur Hospital, Ministry of Health Malaysia, Kuala Lumpur 50586, Malaysia; rosnayahya@gmail.com; 5 Faculty of Pharmacy, Universitas Airlangga, Surabaya 60115, Indonesia

**Keywords:** ABCC2, CYP3A5, kidney transplant recipients, polymorphism, tacrolimus, clinical outcome

## Abstract

Personalized medicine in kidney transplantation has the potential to improve outcomes and reduce complications. The aim of this study was to investigate the influence of single nucleotide polymorphisms in genes encoding metabolizing enzymes (CYP3A5) and transporters (ABCC2) on clinical outcomes (acute graft failure and/or acute tubular necrosis (ATN)) in kidney transplant recipients (KTR). This was a multicenter, retrospective cohort study where adult KTR who had undergone kidney transplantation between 2020 and 2021 and received tacrolimus-mycophenolate treatment were enrolled in the study. DNA was extracted from collected blood samples using a commercially available kit. CYP3A5*3, *ABCC2 -24C>T* and *ABCC2 3972C>T* SNP were determined by polymerase chain reaction. Of the total 39 patients included, nine (23.1%) KTR had an incidence of acute graft failure and/or ATN. A multiple logistic regression showed wildtype *ABCC2 -24C>T C* allele had a higher risk of developing acute graft rejection and/or ATN compared to the variant allele carriers (adjusted Odd Ratios [aOR]: 27.675, *p* = 0.038). Recipients who had delayed graft function (aOR: 49.214, *p* = 0.012) and a history of CMV infection (aOR: 18.097, *p* = 0.009) were at 49.2 and 18.1-times increased risk for acute graft failure and/or ATN, respectively. The large aOR was inevitable due to the small sample size and required cautious interpretation. This is the first study to determine the effect of the *ABCC2 -24C>T* genetic polymorphism on clinical outcomes in Malaysian KTR and forms the basis for further work on *ABCC2 -24C>T* effects in long-term KTR.

## 1. Introduction

Tacrolimus and mycophenolic acid (MPA) remain the cornerstone for immunosuppressive therapy for patients who have undergone kidney transplantation worldwide [1]. Both drugs exhibit high intra-individual and inter-individual variability in their pharmacokinetic profile [1]. Although tacrolimus and MPA are the gold standard drugs for kidney transplant recipients (KTR), the risk of adverse drug reactions (ADR) and rejection during the use of immunosuppressants continues to be a major clinical problem. Apart from medication adherence, achieving targeted drug levels and managing ADR to ensure the safe use of immunosuppressive therapy among KTR [2], recent work has demonstrated that other factors such as genetic variability derived from single nucleotide polymorphisms (SNPs) may also contribute to the efficacy of drug therapy [3]. Tacrolimus is extensively metabolized by CYP3A5 in the liver and small intestine [4] and is a substrate of P-glycoprotein (P-gp), encoded by the multidrug resistance *(MDR) 1/ABCB1* gene [5]. MPA is glucuronidated by uridine diphosphate-glucuronosyltransferases (UGTs) to its pharmacologically inactive 7-O-glucuronide metabolite (MPAG) [6], to which MPAG is then excreted into the bile via the multidrug resistance-associated protein-2 (MRP2/ABCC2) that is encoded by the *ABCC2* gene [7]. The MRP2 transporter encoded by the *ABCC2* gene may also be involved in the clearance of tacrolimus [8]. Therefore, drug molecules of tacrolimus are metabolized by CYP3A5 prior to reaching the systemic circulation, whereas the MRP2/ABCC2 transporter is involved in the enterohepatic recirculation of MPA and in tacrolimus clearance. This has led to the possibility that genetic polymorphisms within *CYP3A5* and *MRP2*/*ABCC2* could possibly account for a significant part of the inter-individual variability observed with tacrolimus bioavailability and MPA exposure, as well as the diverse therapeutic and adverse effects of the drugs observed among patients.

The best-studied genetic variation is in the *CYP3A5* gene [9,10], which is the predominant enzyme for the metabolism of tacrolimus, with a higher affinity in comparison with *CYP3A4* [11]. It has been consistently demonstrated that *CYP3A5* expressers require at least a 1.5-fold higher tacrolimus dose compared with *CYP3A5* non-expressers to reach the same exposure [12]. Despite this, only a few studies have been conducted locally to investigate the effects of the *CYP3A5* polymorphism on tacrolimus pharmacokinetics [13,14] and there have been no findings beyond drug levels examining the outcomes of transplantation. Compared to European countries, data on genetic variation in genes transcribing drug metabolizing enzymes among Asians, such as *CYP3A5* is limited due to ethnic diversity, thus a need for pharmacogenetic studies among Asians to better understand the gene–drug interaction among this population [15].

On the other hand, results on the impact of *ABCC2* SNP towards MPA pharmacokinetics have been conflicting, with studies confirming the impact of the MRP2 *C-24T* polymorphism on MPA pharmacokinetics, in which SNP is associated with a lower oral clearance of MPA in steady-state conditions [16]. This is in contrast to a study that concluded that polymorphisms in *ABCC2 C-24T* showed no consistent effect on MPA pharmacokinetics and toxicity [17]. Interestingly, it was also documented that patients with the *MRP2* SNP reported more diarrhea in the first year after transplantation [16] while another study demonstrated no significant effect of *ABCC2* SNPs or haplotype on the risk of diarrhea in the study population [18], demonstrating possible effects of gene variations of ADR. No further studies were conducted examining its SNP effect on the overall transplantation outcomes.

The inter-individual variations in activity and expression levels of *CYP3A5* and *ABCC2* due to SNP may affect the therapeutic effect and ADR of the immunosuppressive regimen as well as the overall transplant outcome among KTR. Data on the association of genetic variants of *CYP3A5* and *ABCC2* genes with kidney transplant outcomes are scarce, especially among Asians. Therefore, the purpose of this study was to investigate the association of genetic polymorphisms (CYP3A5*3, *ABCC2 -24C>T* and *ABCC2 3972 C>T*) in modulating the clinical outcome of transplantation among Malaysian KTR. 

## 2. Materials and Methods

### 2.1. Study Subjects

A multicenter, retrospective cohort study involving adult KTR who had undergone kidney transplantation between the years 2020 and 2021 was conducted in two major kidney transplant centers in Malaysia: General Hospital, Kuala Lumpur and Selayang Hospital, Selangor, Malaysia. Genetic samples were collected once potential patients were identified via screening of patient lists and data from medical records. Written informed consent was obtained from every subject prior to their participation in this study. Patients were recruited if they were taking tacrolimus-MPA immunosuppressive agents for at least 12 months following kidney transplantation. Patients who were pregnant, lactating and had incomplete clinical information were excluded.

The study protocol was approved by the Universiti Kebangsaan Malaysia Research Ethics Committee (UKM PPI/111/8/JEP-2022-431) and registered under the National Medical Research Register, Ministry of Health Malaysia (NMRR ID-22-00076-D3H (IIR)). This study was conducted in compliance with ethical principles outlined in the Declaration of Helsinki and the Malaysian Good Clinical Practice Guidelines. The study report follows the Strengthening the Reporting of Pharmacogenetic Studies (STROPS) guidelines [19]. 

### 2.2. Data Collection

After obtaining written informed consent from subjects, a 5 ml blood sample was collected in two separate tubes. Baseline demographic data, clinical information, laboratory results and medication intake for 1 year after transplantation were collected from the hospital’s electronic medical record and chart review. 

The demographic data collected include age, gender, ethnicity and body weight. The clinical information was composed of pre-transplant variables namely primary diagnosis, co-morbidities, blood pressure (BP) reading prior to transplant, dialysis modality, dialysis duration and types of transplants. Details of the concomitant medication regimen were noted. For medications taken prior to transplant, only the total number of medications taken was recorded for each patient. Patient kidney function denoted as estimated glomerular filtration rate (eGFR) was calculated using the Chronic Kidney Disease Epidemiology Collaboration (CKD-EPI) equation [20]. Body weight, BP reading, tacrolimus daily dose and its corresponding tacrolimus trough level, as well as MPA doses were recorded. Other data included were the presence of delayed graft function (DGF) defined as the need for dialysis within the first week after transplantation [21], acute rejection diagnosed as biopsy-proven acute rejection (BPAR) or clinical acute rejection, calcineurin inhibitor (CNI) toxicity, chronic allograft nephropathy (CAN), post-transplant diabetes mellitus (PTDM) and hypertension, hospital admission due to infection, transaminitis, diarrhea, malignancy, CMV infection, BKV infection, urinary tract infection (UTI), acute tubular necrosis (ATN) and leukopenia.

### 2.3. DNA Extraction and SNP Genotyping 

Peripheral blood samples were collected from the study subjects after obtaining informed consent, and the genomic DNA was extracted using a Qiagen DNeasy blood and tissue extraction kit (Qiagen, Hilden, Germany) in accordance with the manufacturer’s DNA extraction protocol. The extracted genomic DNA was then analyzed for purity [22]. 

The *CYP3A5* polymorphism *12083A>G* and *ABCC2* polymorphisms *-24C>T* and *3972C>T* genotyping was conducted with a polymerase chain reaction (PCR) that was segregated by size and captured using the E-Gel^®^ Safe Imager^TM^ Realtime Transilluminator (Life Technologies, Kiryat Shmona, Israel) [23]. The PCR product was then purified by a commercialized PCR purification kit (Applied Biosystems, Warrington, UK) as a precondition for DNA Sanger sequencing. Purified DNA fragments were analyzed using the BigDye^®^ Terminator version 3.1 cycle sequencing kit, which was run on a 96-capillary 3730xl DNA Analyzer at First BASE Laboratories Sdn. Bhd., Malaysia (developed by Applied Biosystem, Waltham, MA, USA, and produced by Thermo Fisher Scientific, Waltham, MA, USA). The DNA sequences of the SNP results were transcribed using Sequence Scanner version 2.0 software (Applied Biosystems) and checked with the reference sequences in the Basic Local Alignment Search Tool (BLAST) program to confirm the presence of the polymorphism [24]. Three DNA samples obtained from the subjects’ pool were randomly genotyped and sent for direct sequencing. Sequencing results confirmed the validity of the method. 

### 2.4. Clinical Outcome

Primary clinical outcomes were patient survival, graft survival and factors affecting primary clinical outcomes. Patient survival was defined by the time from transplant to patient death or the date of the last follow-up [25]. Graft survival was defined as (i) graft survival without acute graft rejection and/or ATN was survival of transplant without the return to long-term dialysis, graft nephrectomy and re-transplantation and/or diagnosis of ATN [25], or, (ii) graft survival with acute graft rejection and/or ATN was defined as graft survival with an incidence of acute graft rejection (diagnosed as biopsy proven acute rejection or clinical acute rejection) and/or diagnosis of ATN but without the return to long-term dialysis, graft nephrectomy and re-transplantation.

Secondary clinical outcomes were defined as clinical observations and ADR. Clinical observations refer to the common parameters that were routinely monitored during the patient’s outpatient clinic follow-up and included the recipient’s kidney function denoted by serum creatinine and eGFR calculated using the CKD-EPI equation, body weight, BMI, BP and the presence of DGF. ADR observed in this study included acute rejection, diagnosed as BPAR or clinical acute rejection, CNI toxicity, CAN, PTDM and hypertension, hospital admission due to infection, transaminitis, diarrhea, malignancy, CMV infection, BKV infection, UTI, ATN and leukopenia. ADRs were noted in patient files as assessed by clinicians or pharmacists at the point of care. The ADRs were independently assessed by two pharmacists and possible ADRs were included in the study.

### 2.5. Statistical Analysis

Descriptive statistics were used for the characteristics of the study cohort. Continuous variables are reported as means ± standard deviation (SD) for normally distributed data and medians ± ranges if non-normally distributed data and compared with *t*-test or Mann–Whitney U test. Counts and percentages were calculated for categorical data. Patient characteristics were defined as possible determinants of patient and graft survival. Three SNPs in two genes encoding the metabolizing enzyme and MPA transporter were included in this analysis. Adherence of the genotype groups to the Hardy–Weinberg equilibrium (HWE) assumption was examined and assumptions were statistically met. Expected percentages for each genotype group were calculated based on the Hardy–Weinberg equation using the allele frequencies (p2 + 2pq + q2 = 1) [23]. A simple and multiple logistic regression was performed to determine factors associated with graft survival. Bonferroni correction was not applied due to the exploratory observational nature of the study, and the results reported were nominal *p*-values that have not been corrected for multiple hypothesis testing. A *p*-value < 0.05 was considered statistically significant.

## 3. Results

### 3.1. Patient Characteristics

A total of 39 KTR were included in this study, of which 21 (53.8%) were males. Demographic data and clinical information on KTR are shown in Table 1. The recipients had a mean age of 32.2 ± 7.0 at the time of transplant and the population was predominantly Malay ethnicity (*n* = 25, 64.1%). The majority of the recipients were diagnosed with glomerulonephritis (*n* = 18, 46.2%) as the primary disease that caused end-stage kidney disease followed by unknown causes (*n* = 17, 43.6%), as many of them were already needing long-term dialysis by the time they were referred to nephrologists for further treatment. The average pre-transplant dialysis duration was 62.1 ± 60.3 months. Hypertension (*n* = 30, 76.9%) was the most common pre-existing condition among the KTR. Most patients received induction therapy with basiliximab (*n* = 35, 89.7%) and MMF (*n* = 26, 66.7%) as the antiproliferative agent.

### 3.2. Clinical Outcomes

The primary outcomes showed that the overall patient and graft survival were 100% at year 1 (Table 2). Despite 100% graft survival, 23.1% (*n* = 9) of the subjects experienced biopsy-proven acute graft rejection and ATN.

The secondary outcomes in Table 2 were assessed and demonstrated that the serum creatinine values steadily improved from 224.0 µmol/L (interquartile range, IQR 130.3–407.8 µmol/L) on day 7 at baseline to 128.5 µmol/L (IQR 93.5–143.8 µmol/L) on 12-month following kidney transplantation, with the corresponding eGFR readings that increased from 37.3 ± 26.8 mL/min/1.73 m^2^ to 60.3 ± 16.8 mL/min/1.73 m^2^.

On day 7 (baseline) after kidney transplantation, the mean trough concentration of tacrolimus was 7.2 ± 2.6 ng/mL, which was lower than the recommended target trough of 8–10 ng/mL and 10–12 ng/mL for low and high immunological risk, respectively, for the first 2 months post-transplant [26]. In contrast, the mean trough concentration of tacrolimus was 6.7 ± 1.5 ng/mL at month 12, which was higher than the recommended target trough for low immunological risk patients, which is 4–6 ng/mL, but within the recommended range for high immunological risk patients, which is 6–8 ng/mL [26].

Among the ADR experienced by KTR, post-transplant hypertension was reported to be the highest (*n* = 16, 41.0%), followed by a history of admission due to infection (*n* = 9, 23.1%) and diarrhea suffered by eight KTR (20.5%) (Table 2).

### 3.3. Pharmacogenetic Analysis

The genotype or allele frequencies of CYP3A5*3, *ABCC2 -24C>T* and *ABCC2 3972C>T* were analyzed in the 39 KTR maintained with tacrolimus-MPA-prednisolone immunosuppressive therapy. The frequencies of CYP3A5*3, ABCC2 -24C>T and *ABCC2 3972C>T* variant alleles were 0.641, 0.205 and 0.282 in the whole patient cohort, respectively. CYP3A5 *1/*1, CYP3A5 *1/*3 and CYP3A5 *3/*3 genotypes were detected in 12.8% (*n* = 5), 46.2% (*n* = 18) and 41% (*n* = 16), respectively. For *ABCC2 -24C>T* and *ABCC2 3972C>T*, the *ABCC2 -24C>T CC, CT* and *TT* genotypes were detected in 59% (*n* = 23), 41% (*n* = 16) and zero recipients, respectively. The *3972C>T CC, CT* and *TT* were found in 46.2% (*n* = 18), 51.6% (*n* = 20) and 2.5% (*n* = 1). The genotype distribution for all polymorphisms investigated was in accordance with the Hardy–Weinberg equilibrium [23].

### 3.4. Factors Associated with Graft Survival in the Presence of Acute Graft Rejection and/or ATN

To determine factors that influence the development of graft survival with the presence of acute graft rejection and/or ATN, a simple logistic regression analysis was first performed. Risk factors for the development of graft survival with the presence of acute graft rejection and/or ATN included the genetic polymorphisms studied, demographic characteristics such as age, baseline BMI and underlying comorbidities as well as transplant-related factors and ADR due to immunosuppressive regimen (Table 3). Genetic factors were included to determine their effects on the model as well as all variables that demonstrated a *p*-value of <0.25 [27]. Following a multiple logistic analysis, the wildtype *ABCC2 -24C>T C* allele (adjusted Odd Ratios [aOR]: 27.675, 95% confidence interval [CI]: 1.204, 636.151), presence of DGF (aOR: 49.214, 95% CI: 2.366, 1023.731) and CMV infection (aOR: 18.097, 95% CI: 2.036, 160.867) resulted in an increased risk of graft survival with complications (Table 3). Patients with the wildtype *ABCC2 -24C>T C* allele tend to have a 27.7 times chance of developing complications compared to the variant allele carriers (*p* = 0.038). Those who had DGF had 49.2 times the chance of developing complications than those without DGF (*p* = 0.012). In addition, KTR with a history of CMV infection were found to be 18.1 times (*p* = 0.009) more likely to develop complications. Multicollinearity and interaction terms were checked and not found, while the model of fitness was satisfactory based on the Hosmer–Lemeshow test (*p* = 0.117) and was able to correctly identify outcomes by 83.3%.

## 4. Discussion

A review of the global success rate in kidney transplantation has highlighted that the long-term benefits from kidney transplantation have not improved significantly in the same magnitude as acute rejection rates and short-term graft survival among KTR [28,29]. Such a limitation may be further compounded by the fact that no new alternative immunosuppressants have been found thus far that can safely replace tacrolimus and MPA as the first-line immunosuppressive agent in kidney transplantation [30]. The current work highlights the importance of the tacrolimus-mycophenolate regimen for KTR with the study cohort reporting an overall graft and patient survival rate of 100% at 1 year following transplantation. This is consistent with other research work that reports that on average, KTR experience 1-year graft and patient survival rates exceeding 95% [31,32]. Among the KTR, the majority were Malays, which is in line with the population of Malaysia. The CYP3A5*3 allele was found to be the predominant allele and the CYP3A5 *1/*3 genotype was the predominant genotype. Among these, the majority were *CYP3A5* expressers. Our findings are similar to those reported in other Malaysian or Asian populations. Hamzah et al. have reported the prevalence of CYP3A5*3 was in a range of 50.0–80.2% in Malaysian KTR [13]. When compared to other ethnic groups, the CYP3A5*3 allele SNP is reported to be more common among Caucasians (90–93%) than Asians (60–73%) [33]. To the best of our knowledge, this is the first Malaysian cohort to report *ABCC2 -24C>T* and *ABCC2 3972C>T* allele and genotype within the Malaysian kidney transplant population. The distribution of *ABCC2 -24C>T CC, CT* and *TT* among the Malaysian KTR population was similar to one report involving Spanish KTR, while for *3972C>T*, the Malaysian population reported a higher *3972C>T CT* as the Spanish was more likely to have a CC genotype compared to our study cohort [34].

Among the ADRs, the majority of the KTR reported hypertension. Among KTR, bringing BP under control is indeed crucial for improved allograft survival and reduced mortality [35]. It is important to recognize that apart from the traditional risk factors contributing to increased cardiovascular risk such as hypertension, immunosuppression medications can also exert a negative effect with CNI-based therapy being one of them, resulting in a significant increase in BP [36]. Another frequent ADR reported was post-transplant infections such as UTI, CMV infection, and BKV infection. The reported rates of BKV and CMV infection in our study cohort are in agreement with the published reports indicating an overall BKV of 19% [37] and a CMV infection rate in KTR of 1.22–72% [38]. In addition to this, UTI represents the most common infection after kidney transplantation [39] and management can be challenging as KTR are often clinically asymptomatic due to immunosuppressive therapy, which may progress to more serious life-threatening infective conditions such as bacteremia and urosepsis [40]. CMV and BKV infections are also considered to be the most common opportunistic infections among KTR [41]. It is inevitable for KTR to be infected with bacterial and viral infections due to long-term maintenance of immunosuppression as well as an increase in the use of potent immunosuppressive drugs [41]. As such, the observed infections necessitate awareness since they can adversely affect graft and patient survival [41].

Interestingly, the determinants of complications associated with graft survival in the study cohort were *ABCC2* polymorphism and a history of DGF and CMV infection. The results showed that the *ABCC2 -24C>T* variant allele carrier was a protective factor against transplant-related complications of acute graft rejection and/or ATN in the present study. There are very few studies that evaluate the influence of *ABCC2* SNP on clinical outcomes, in particular graft rejection among KTR treated with tacrolimus and MPA. However, one study performed among 255 KTR demonstrated that the *ABCC2 -24C>T* variant was associated with a reduced risk for infections [42]. This may be explained by the up-regulation of the ABCC2 transporter that increases the efflux of drugs and infection-causing agents, in a method to protect the tissue from damage [43]. Another study involving 148 Brazilian KTR revealed that patients with the *ABCC2 -24C>T* variant treated with tacrolimus and MPA had significantly more diarrhea in the first year after transplantation [16]. This was said to be attributable to *ABCC2 -24C>T* SNP patients who were associated with significantly higher dose-corrected MPA trough levels to ensure optimal immunosuppression [16]. In light of these findings, more work should be performed to identify the effects of *ABCC2 -24C>T* in influencing patient outcomes such as graft survival. With respect to the *ABCC2 3972C>T* SNP and of CYP3A5*1/*1 or CYP3A5*1/*3 expression, no significant findings were reported in relation to graft survival, although previous work shows an increase in the risk for ADR [42].

Alongside the genetic polymorphism, the current work demonstrated that patients with DGF had an increased risk of developing transplant-related complications. DGF is a common and frequent complication of kidney transplantation that requires dialysis during the first week following transplantation [44]. It is a manifestation of acute kidney injury, and several risk factors have been identified to be involved in the development of DGF such as ischemic-reperfusion injury, deceased donor, the quality of the donated kidney and the clinical conditions of the recipients, for example, chronic dialysis patients or patients who developed hypotension, anesthesia or surgery [45]. Over time, patients with DGF may have suboptimal graft function and survival compared to KTR without DGF, which is a predictor of poor long-term graft survival [46]. Besides the association with long-term graft failure, DGF was also found to be associated with higher rates of rejection in a study that involved 42,736 living donor recipients [46]. This can be explained by the occurrence of ischemia-reperfusion injury during the transplantation process that is manifested as DGF [47]. Reperfusion of the damaged kidney will then augment the injury through complement activation and accumulation of free radicals that result in the activation of antigen-presenting cells which present alloantigens to T-lymphocytes. It is the priming of T-cells that leads to the development of acute rejection among DGF KTR [48].

Another factor associated with graft survival was CMV infection, which has previously been shown to be associated with an increased risk of renal graft rejection, graft loss and mortality [49]. Our results are consistent with Nett PC et al. in demonstrating an increased likelihood of developing acute graft rejection in KTR with a history of CMV infection [50]. Although the exact reason is unclear, one possibility is that the treatment of CMV infection consists of a reduction in immunosuppression that may trigger the occurrence of graft rejection [51]. In addition to this, CMV typically causes tubulointerstitial nephritis which is characterized by inflammation of the renal tubules and interstitium [52], similar to ATN, which is characterized as acute tubular cell injury and dysfunction, generally caused by hypotension, sepsis, or nephrotoxic drugs [53].

To that end, the findings suggest the possible role of determining the patient’s *ABCC2 -24C>T* genotype, as well as DGF and CMV as part of the clinical management to avoid or minimize graft failure among KTR. However, further work is required to confirm these findings in a larger sample size. The large estimate of aOR was inevitable due to an underpowered sample size and requires cautious interpretation. In view of the small number of KTR due to the limited donors within the local community, pharmacogenetic studies can be challenging in the local setting, albeit they are required to ensure the optimal management of patients. The current work, however, does point towards the possible role of genetic screening, which has become much more affordable in the past and could signify a move forward in managing KTR. In addition, other polymorphisms in the genes related to CYP3A4, ABCB1, or the human pregnane X receptor (PXR) are likely important and could be included in future work. Nonetheless, our results should be interpreted with caution and further comprehensive studies should be carried out to confirm our findings. At this point, the current work does form a basis for future studies that may aid in the personalization of drug treatment according to individual recipient’s genetic make-up and alongside other clinical parameters, such as immunological risk, other medications and donor characteristics to achieve potentiation of therapeutic efficacy and avoidance of graft failure.

## 5. Conclusions

In conclusion, the results of our study indicated a relationship between *ABCC2 -24C>T* polymorphisms and genetic dispositions, DGF and CMV to transplant-related complications in KTR treated with tacrolimus and MPA immunosuppressive therapy. The current work forms a basis for the further detection of *ABCC2 -24C>T SNP* and its effects among KTR in Malaysia and may be important as we move towards individualized drug therapy in kidney transplantation. The application of genetic screening could support future personalization of treatment among KTR and may possibly result in the reduction in complications that could lead to improved graft and patient survival as well as quality of life. This could also potentially reduce the overall cost of medical treatment among KTR.

## Figures and Tables

**Table 1:** Demographic and clinical characteristics of participants (*n* = 39).

Parameter	Values
Demographics	
Age at transplant, years, mean ± SD	32.2 ± 7.0
Gender, *n* (%)	
Male	21 (53.8)
Female	8 (20.5)
Ethnicities, *n* (%)	
Malay	25 (64.1)
Chinese	5 (12.8)
Indian	9 (23.1)
Others	0 (0.0)
Clinical	
Total body weight, kg, mean ± SD	60.7 ± 17.4
BMI, kg/m^2^, mean ± SD	22.7 ± 4.2
SBP pre-transplant, mmHg, mean ± SD	142.9 ± 19.7
DBP pre-transplant, mmHg, mean ± SD	90.8 ± 12.2
Primary renal disease, *n* (%)	
Autosomal dominant polycystic kidney disease	1 (2.6)
GN	18 (46.2)
Diabetic nephropathy	1 (2.6)
Hypertensive	1 (2.6)
Lupus nephritis	1 (2.6)
Unknown	17 (43.6)
Comorbidities, *n* (%)	
Hypertension	30 (76.9)
Both hypertension and diabetes	3 (7.7)
Others	15 (38.5)
Dialysis	38 (97.4)
Preemptive	1 (2.6)
Dialysis modality, *n* (%)	
PD	6 (15.4)
HD	25 (64.1)
Mix mode	
PD to HD	5 (12.8)
HD to PD	2 (5.1)
Duration of dialysis, months, mean ± SD	62.1 ± 60.3
Type of donor, *n* (%)	
Living-related	30 (76.9)
Cadaveric	9 (23.1)
Induction treatment, *n* (%)	
Basiliximab	35 (89.7)
ATG	4 (10.3)
Average number of medications taken prior to transplant, mean ± SD	6.5 ± 2.2
Average number of medications other than ISA following transplant, mean ± SD	4.0 ± 2.0

Abbreviations: ATG, antithymocyte globulin; BMI, body mass index; DBP, diastolic blood pressure; GN, glomerulonephritis; HD, hemodialysis; ISA, immunosuppressive agent; PD, peritoneal dialysis; SBP, systolic blood pressure; SD, standard deviation.

**Table 2:** Clinical outcomes at month 12 among post-kidney transplant recipients within the study group (*n* = 39).

Variables	*n* (%)
Primary clinical outcomes	
Patient survival, *n* (%)	39 (100)
Graft survival, *n* (%)	39 (100)
with incidence of rejection and ATN, *n* (%)	9 (23.1)
without incidence of rejection and ATN, *n* (%)	30 (76.9)
Secondary clinical outcomes	
Serum creatinine, µmol/L, median (IQR)	
Day-7 (baseline)	224.0 (130.3–407.8)
12-Month	128.5 (93.5–143.8)
eGFR, mL/min/1.73 m^2^, mean ± SD	
Day-7 (baseline)	37.3 ± 26.8
12-Month	60.3 ± 16.8
C_0_ of tacrolimus, ng/mL, mean ± SD	
Day-7 (baseline)	7.2 ± 2.6
12-Month	6.7 ± 1.5
Body weight, kg, mean ± SD	64.8 ± 14.5
BMI, kg/m^2^, mean ± SD	23.7 ± 4.5
SBP	125 ± 13.9
DBP	78.9 ± 9.9
DGF, *n* (%)	
Yes	6 (15.4)
No	33 (84.6)
ADR presence, *n* (%)	
Acute graft rejection	2 (5.1)
CNI toxicity	1 (2.6)
CAN	0 (0.0)
PTDM	1 (2.6)
Post-transplant hypertension	16 (41.0)
History of hospital admission for infection	9 (23.1)
Transaminitis	7 (17.9)
Diarrhea	8 (20.5)
Malignancy	0 (0.0)
CMV infection	7 (17.9)
BKV infection	2 (5.1)
UTI	8 (20.5)
ATN	7 (17.9)
Leukopenia	2 (5.1)

Abbreviations: ADR, adverse drug reaction; ATN, acute tubular necrosis; BKV, BK virus; BMI, body mass index; C_0_, trough concentration, CMV, cytomegalovirus; CNI, calcineurin inhibitor; CAN, chronic allograft nephropathy; DBP, diastolic blood pressure; DGF, delayed graft function; eGFR, estimated glomerular filtration rate; IQR, interquartile range; PTDM, post-transplant diabetes mellitus; SBP, systolic blood pressure; SD, standard deviation; UTI, urinary tract infection.

**Table 3:** Factors associated with graft survival in the presence of acute graft rejection and/or ATN (inclusive of all alleles).

**Simple Model**					
**Variables (Ref)**	**B**	**OR**	**95% CI**		***p*-Value**
Genetic polymorphisms					
CYP3A5*1 allele (*3 variant)	0.147	1.158	0.381	3.520	0.796
*ABCC2 -24C>T* C allele (T variant)	0.089	2.435	0.498	11.904	0.272
*ABCC2 3972C>T* C allele (T variant)	0.028	1.028	0.318	3.326	0.963
Demographics					
Age (years)	0.044	1.045	0.941	1.160	0.410
Male (female)	2.348	10.462	1.158	94.482	0.037
Ethnicity (Malay)					
Chinese	0.981	2.667	0.347	20.508	0.346
Indian	−0.693	0.500	0.050	4.978	0.554
Clinical pre-transplant					
BMI, kg/m^2^	−0.005	0.995	0.815	1.215	0.962
SBP, mmHg (<130)	−0.118	0.889	0.081	9.767	0.923
DBP, mmHg (<80)	−0.134	0.875	0.143	5.337	0.885
Primary renal disease (GN)					
Others ^a^	0.223	0.125	0.101	15.499	0.862
Unknown	0.223	0.125	0.257	6.07	0.782
Comorbidities (No)					
Hypertension	−0.223	0.800	0.121	5.269	0.817
Both hypertension and diabetes	0.693	2.000	0.115	34.822	0.634
Others	1.386	4.000	0.167	95.756	0.392
Dialysis (Preemptive)	20.033	0.000	0.000		1.000
Dialysis modality (HD)					
PD	−0.665	0.514	0.051	5.221	0.574
Mixed mode	−0.847	0.429	0.043	4.232	0.468
Duration of dialysis, months	0.007	1.007	0.995	1.019	0.008
Type of donor, Living-related (Cadaveric)	−1.386	0.250	0.049	1.274	0.095
Inductive treatment, basiliximab (ATG)	−1.386	0.250	0.030	2.099	0.202
Average number of medications taken prior to transplant	−0.129	0.879	0.618	1.249	0.472
Average number of medications other than ISA following transplant	0.430	1.538	1.006	2.351	0.047
Clinical post-transplant					
eGFR at 12-month, mL/min/1.73 m^2^ (>40)	1.386	4.000	0.476	33.585	0.202
Tacrolimus trough level up, ng/mL (within range 4-8)	0.134	1.143	0.187	6.971	0.885
BMI at 12-month, kg/m^2^ (<24.9)	−0.147	0.864	0.179	4.161	0.855
SBP at 12-month, mmHg (<130)	−0.147	0.864	0.179	4.161	0.855
DBP at 12-month, mmHg (<80)	−0.045	0.956	0.213	4.284	0.953
DGF (No)	2.416	11.200	1.600	78.400	0.015
ADR (No)					
Post-transplant hypertension	0.984	2.676	0.475	15.089	0.265
History of hospital admission for infection	−0.063	0.939	0.158	5.593	0.945
Transaminitis	0.357	1.429	0.227	9.009	0.704
Diarrhea	0.916	2.500	0.463	13.495	0.287
CMV infection	1.179	3.250	0.570	18.523	0.184
UTI	0.134	1.143	0.187	6.971	0.885
Others ^b^	0.619	1.857	0.28	12.311	0.521
**Multiple Logistic Model**					
**Factors (Ref)**	**B**	**Adjusted OR**	**95% CI**		***p*-Value**
Genetic polymorphisms					
CYP3A5*1 allele (*3 variant)	0.820	2.270	0.314	16.399	0.416
*ABCC2 -24C>T* C allele (T variant)	3.321	27.675	1.204	636.151	0.038
*ABCC2 3972C>T* C allele (T variant)	−2.523	0.080	0.005	1.284	0.075
Demographics					
Male (female)	2.105	8.207	0.811	83.086	0.075
Clinical pre-transplant					
Duration of dialysis, months	0.011	1.008	0.983	1.033	0.548
Type of donor, Living-related (Cadaveric)	0.571	1.770	0.031	100.193	0.782
Inductive treatment, basiliximab (ATG)	0.472	1.604	0.091	28.322	0.747
DGF (No)	3.896	49.214	2.366	1023.731	0.012
Average number of medications other than ISA following transplant	0.094	1.098	0.685	1.760	0.698
Clinical post-transplant					
eGFR at 12-month, mL/min/1.73 m^2^ (>40)	−0.908	0.403	0.029	5.615	0.499
CMV infection (No)	2.896	18.097	2.036	160.867	0.009

Abbreviations: ADR, adverse drug reaction; ATG, antithymocyte globulin; BMI, body mass index; CMV, cytomegalovirus; DBP, diastolic blood pressure; DGF, delayed graft function; eGFR, estimated glomerular filtration rate; GN, glomerulonephritis; HD, hemodialysis; ISA, immunosuppressive agent; PD, peritoneal dialysis; SBP, systolic blood pressure. ^a^ Other primary renal diseases include lupus nephritis, type 1 diabetes mellitus, polycystic kidney disease. ^b^ Other ADR include CNI toxicity, PTDM, BKV, leukopenia.

## Data Availability

Authors do not have permission to share the data. The data underlying the results presented in the study are available upon request from the corresponding author (faridaislahudin@ukm.edu.my) for researchers who meet the criteria for access to confidential data.
